# Impact of ionomers on porous Fe-N-C catalysts for alkaline oxygen reduction in gas diffusion electrodes

**DOI:** 10.1038/s42004-025-01422-4

**Published:** 2025-01-31

**Authors:** Jinjie Zhu, Angus Pedersen, Simon Kellner, Robert D. Hunter, Jesús Barrio

**Affiliations:** 1 Department of Chemical Engineering, Imperial College London, London, SW7 2AZ UK; 2 Department of Materials, Royal School of Mines, Imperial College London, London, SW7 2AZ UK

**Keywords:** Electrocatalysis, Fuel cells

## Abstract

Alkaline exchange membrane fuel cells (AEMFCs) offer a promising alternative to the traditional fossil fuel due to their ability to use inexpensive platinum group metal (PGM)-free catalysts, which could potentially replace Platinum-based catalysts. Iron coordinated in nitrogen-doped carbon (Fe-N-C) single atom electrocatalysts offer the best Pt-free ORR activities. However, most research focuses on material development in alkaline conditions, with limited attention on catalyst layer fabrication. Here, we demonstrate how the oxygen reduction reaction (ORR) performance of a porous Fe-N-C catalyst is affected by the choice of three different commercial ionomers and the ionomer-to-catalyst ratio (I/C). A Mg-templated Fe-N-C is employed as a catalyst owing to the electrochemical accessibility of the Fe sites, and the impact of ionomer properties and coverage were studied and correlated with the electrochemical performance in a gas-diffusion electrode (GDE). The catalyst layer with Nafion at I/C = 2.8 displayed the best activity at high current densities (0.737 ± 0.01 V_RHE iR-free_ at 1 A cm⁻²) owing to a more homogeneous catalyst layer, while Sustainion displayed a higher performance in the kinetic region at the same I/C. These findings provide insights into the impact of catalyst layer optimization to achieve optimal performance in Fe-N-C based AEMFCs.

## Abstract (web summary)

Fe-N-C single atom electrocatalysts offer high catalytic activities for the oxygen reduction reaction, but limited attention has been paid to the morphology of the catalyst layers. Here, the authors demonstrate the impact of three different commercial ionomers on the electrocatalytic performance of porous Fe-N-C layers in alkaline media.

## Introduction

Green hydrogen-supplied fuel cells stand as a promising alternative to traditional fossil fuel-derived power generation due to their environmentally friendly properties and high efficiency. However, the sluggish oxygen reduction reaction (ORR) requires platinum group metal (PGM)-based catalysts, which are associated with high cost and scarcity. Owing to the enhanced oxygen reduction reaction (ORR) kinetics of PGM-free catalysts in the alkaline environments of anion exchange membrane fuel cells (AEMFCs) compared to Proton exchange membrane fuel cells (PEMFCs)^1^, their performance can equal that of PGM catalysts, which can reduce the cost barrier to the commercialization of AEMFCs^2^. Iron coordinated to nitrogen-doped carbon (Fe-N-C) single atom catalysts stand as the best alternative to traditional Pt nanoparticle-based catalysts for oxygen reduction due to their high intrinsic activity and selectivity to the 4e^-^ pathway^3^. While the ORR performance of Fe-N-C catalysts has been widely studied in the literature, most of these studies rely on the rotating disk electrode (RDE) for evaluating catalyst activity in both acidic and alkaline environments^4,5^. However, discrepancies exist between the ORR performance measured using RDE and that observed in membrane electrode assemblies (MEA) employed in fuel cell systems^6^. These differences arise due to the variations in electrolytes (liquid versus solid), the composition of the catalyst layer^7^, and the interactions between the catalyst, catalyst support, and ionomer, which significantly influence mass transport during the ORR. Since RDE technology for ORR is limited to low current densities and idealized catalyst layers, it cannot accurately assess the impact of catalyst layer parameters on ORR performance. Additionally, the direct study of AEMFCs is complex, requiring consideration of issues such as water management and difficulty of isolating cathodic response due to lack of a reference electrode^8^. The development of gas-diffusion electrodes (GDE) allows testing environments to better mimic the actual operating conditions of MEAs with less complexity, making GDEs a valuable tool for bridging the gap between fundamental and applied fuel cell catalyst research^9^.

Most research on GDEs has focused on Pt-based catalysts in acidic environments; Arenz and co-workers studied the effect of ink formulation, particularly the ratio of carbon to Nafion, using different commercial Pt/C catalysts in GDE setups^10^. A similar study was later conducted by Cherevko and co-workers, which examined the effect of the ionomer-to-catalyst ratio (*I*/*C*) using a commercial Pt/C catalyst (HiSPEC4000) and identified an optimal value of 0.7^11^. Recently, Mazzucato et al. investigated the impact of various experimental parameters, such as catalyst loading and pH, on the electrocatalytic activity and selectivity of a Fe-N-C catalyst derived from Vulcan XC72 carbon black and Fe(phen)_3_Cl_2_ in both RDE and GDE setups^12^. Mazzoli et al. developed a xylose-derived Fe-N-C catalyst and compared its performance in an alkaline GDE to that of a commercial Fe-N-C (Pajarito PMF-D14401)^13^. Ku et al. used GDE half-cell coupled with inductively coupled plasma mass spectrometry to study the degradation mechanism of Fe-N-C and quantify the Fe dissolution rates in an alkaline medium^14^. So far, however, the impact of the type and amount of anion exchange ionomers on Fe-N-C performance under practical current densities has remained unexplored. Kellner et al. recently investigated how different combinations of three commercial alkaline exchange ionomers and membranes affect the ORR performance of a commercial Fe-N-C catalyst (Pajarito PMF-D14401) in a GDE setup. Their findings demonstrated that the combination of Piperion ionomer and Sustainion membrane delivered the highest ORR performance at 2 A cm⁻² (0.732 V_RHE_). However, the study maintained a constant *I*/*C* throughout, which may not be optimal for all types of ionomers^15^. Additionally, although PMF-D14401 stands as a high-performing benchmark material, its active site density obtained via nitrite stripping is the lowest observed among those reported (0.25 × 10¹⁹ sites g⁻¹)^16^. Although there have been studies related to Pt/C catalysts, such as the work by Hyun et al. on the interaction between ionomer and carbon in AEMFC using four different alkaline ionomers and Nafion with Pt/C catalysts^17^, it remains uncertain whether the findings on Pt/C can be applied to Fe-N-C catalysts.

Therefore, in this work, we study the GDE performance of a recently developed Fe-N-C catalyst with a high micro and mesopore volume that provides a high electrochemical exposure of the FeN_x_ active sites^18^. The material, prepared by pyrolysis of 2,4,6-Triaminopyrimidine (TAP) and MgCl_2_.6H_2_O followed by Fe-coordination, is characterized by means of X-ray diffraction (XRD), N_2_ sorption, and X-ray photoelectron spectroscopy (XPS) and the electrocatalytic activity initially screened in alkaline electrolyte via RDE measurements. The catalyst is then sprayed on carbon electrodes employing different ionomers (namely Sustainion, Piperion, or Nafion) and *I*/*C* ratios, and its GDE performance is assessed in alkaline electrolyte, where the performance was correlated to catalyst layer morphology, as well as ionic hydroxide conductivity.

## Results and discussions

**Fig. 1:**
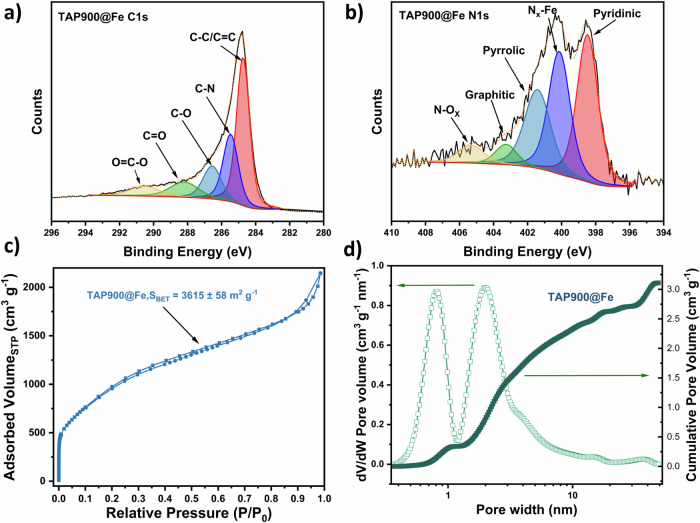
Characterization of the catalyst. **a** C1s and (**b**) N1s XPS spectra of TAP900@Fe. **c** N_2_ sorption isotherms with specific BET surface areas. **d** Pore size distributions and cumulative pore volumes calculated using 2D-non-local density functional theory (NLDFT) heterogeneous surface carbon model in SAIEUS software of TAP900@Fe.

The catalysts were prepared by pyrolysis of TAP and porogen with MgCl_2_·6H_2_O at 900 °C followed by transmetalation with FeCl_2_ through a methanol reflux. This synthetic approach allows the formation of electrochemically accessible FeN_x_ sites in a highly porous nitrogen-doped carbon scaffold^18^. XRD measurements were first conducted to screen whether Fe-based aggregates remained in the material after the metal coordination synthetic step. According to the XRD patterns of TAP900, and TAP900@Fe shown in Supplementary Fig. 1, both samples show a highly amorphous structure with broad carbon peaks, and no diffraction peaks corresponding to Fe oxides, nitrides, carbides, or metallic Fe nanoparticles were observed, suggesting that Fe is present as FeNx moieties, in agreement with our previous results^18^. XPS measurements were further conducted on TAP900@Fe to evaluate the surface chemical composition (Supplementary Table 1) and oxidation states of C and N elements. As shown in Fig. 1a, the C1s spectra of TAP900@Fe show five different binding energies corresponding to structural C-C at 284.8 eV, C-N at 285.5 eV, C-O at 286.6 eV, C=O at 288.2 eV, and O-C=O at 290.6 eV^19^. N1s spectra (Fig. 1b) show a peak corresponding to the N-Fe bond (400.2 eV), as well as peaks at 398.5 eV, 401.5 eV, 403.3 eV, and 405.5 eV that can be assigned to pyridinic, pyrrolic, graphitic, and N-O_x_ nitrogen environments, respectively^20,21^. Additionally, the Fe2p XPS spectra confirm the presence of Fe within the material (Supplementary Fig. 2). For further insights on the active site coordination environment, we would like to refer the reader to our recent work, where X-ray absorption, Mossbauer spectroscopy, and high-angle annular dark-field scanning transmission electron microscopy were employed to unambiguously confirm the atomic dispersion of the TAP-based catalysts^18,22^.

The final material TAP900@Fe displays a Brunauer-Emmett-Teller (BET) specific surface area of 3615 ± 58 m² g^-1^ (Fig. 1c) based on N_2_ sorption measurements. The large initial uptake is attributed to micropores and a further increase due to mesoporosity, suggesting the successful application of the Mg^2+^ porogen^23^. This is confirmed by the pore size distribution results shown in Fig. 1d, with the pore volume centered at pore diameters of 0.8 and 2 nm and subsequent abundant pore volume within the mesopore range. This material exhibits the highest specific surface area among all other Mg-derived catalysts reported in the literature (Supplementary Table 2). Additionally, the material demonstrates a high total pore volume of 3.11 cm³ g⁻¹ and a mesopore/macropore volume of 2.87 cm³ g⁻¹. The active site density in mass or area obtained recently by our group via nitrite stripping of the material^18^ is also compared with those of other Fe-N-C catalysts reported in the literature as shown in Supplementary Table 3, Supplementary Fig. 3, and Supplementary Note 1.

Prior to GDE measurements, the electrochemical oxygen reduction performance of TAP900@Fe was initially screened in a three-electrode system with O₂-saturated 0.1 M KOH electrolyte. Nafion was selected as the ionomer for its ability to maintain the mechanical stability of the catalyst layer and because it is commonly used in RDE studies with alkaline electrolytes^24,25^. Sustainion and Piperion ionomers were also used, but catalyst layers drop-casted onto the electrode with either of them failed to maintain mechanical stability when contacting the electrolyte. Osmieri et al. reported that the use of Piperion alone in the catalyst layer of the AEM electrolyzer led to catalyst detachment, while the combined use of Piperion and Nafion, which is known to have good binding properties, gave structural stability to the catalyst^26^. However, in our case, likely due to the high porosity and surface area of the catalyst, the addition of Nafion (binder-to-ionomer ratio (*B*/*I*) of 0.16) to the Sustainion/Piperion-based inks did not prevent the catalyst detachment completely when exposed to 0.1 M KOH. The addition of a Nafion binder does improve the stability of the catalyst film, with Sustainion showing only small fragments detaching, highlighted in yellow circles (Supplementary Fig. 4b). However, adding a binder to the ink with Piperion did not prevent detachment (Supplementary Fig. 4d), as also highlighted in yellow. Further RDE tests were conducted under the same experimental protocols (Supplementary Fig. 5). Samples with the alkaline ionomer and Nafion binder exhibited higher kinetic current densities compared to those without Nafion, with kinetic current density values of 2.22 mA cm⁻² and 0.32 mA cm⁻² for Sustainion-Nafion and just Sustainion at 0.8 V_RHE_, respectively, aligning with the previous observations. Piperion exhibited severe catalyst detachment during RDE testing (Supplementary Fig. 4c), resembling the performance of pure glassy carbon electrodes. In contrast, samples with Sustainion only showed reduced catalyst detachment under rotation (Supplementary Fig. 4a), achieving a kinetic current density of 0.32 mA cm⁻², compared to 0.005 mA cm⁻² for Piperion at 0.8 V_RHE_. Due to the catalyst detachment, the catalyst loading would be inaccurate, and therefore, the mass activity could not be determined. Therefore, focusing on TAP900@Fe with Nafion ionomer only, the catalyst demonstrated a kinetic mass activity of 0.49 ± 0.07 A g_FeNC_⁻¹ at 0.9 V_RHE_ and 42.1 ± 10.1 A g_FeNC_⁻¹ at 0.8 V_RHE_ (Supplementary Fig. 6), which arises from an outer-sphere electron transfer^27^.

For the GDE testing, a high *I*/*C* of 2.1 and 2.8 was chosen due to the highly porous nature of the catalyst (Fig. 1c), which is expected to provide adequate ionomer coverage, based on previous work^28,29^. The electrolyte concentration was fixed at 1 M KOH for easier comparison with literature^13,14,30^. Additionally, the catalyst loading was chosen to be 0.49 ± 0.02 mg_cat_ cm⁻² for two reasons. Firstly, to maintain relevance with the typical AEMFC catalyst loading using platinum, which ranges from 0.4 to 0.8 mg_ca_ cm⁻² (ref. ^31^). Secondly, The catalyst’s high porosity results in a low volumetric density^28^, leading to thick catalyst layers and nonuniformity of the membrane from the punch-out electrode (see Experimental Methods). In terms of anion exchange ionomers, Sustainion is commercially available and can provide high hydroxyl conductivity (≥100 × 10^−3^ S cm^−1^) to support high current with low resistance losses and maintain stability in KOH electrolyte^32–34^. Piperion is another commercially available ionomer; however, it suffers from weak carbon-ionomer interaction (11.5 kcal mol^−1^ vs. 20.4 kcal mol^−1^ for Sustainion), based on density functional theory calculations^17^. Subsequently, we found TAP900@Fe with Piperion can easily detach from the GDL when contacting the electrolyte, and therefore the ionomer activation was unsuccessful, matching the previous report by Hyun et al.^17^ However, catalyst detachment is not observed when using TAP900@Fe with Sustainion in GDE, and therefore, this work focuses on comparing Sustainion and Nafion ionomers.

Galvanostatic steps with in-situ electrochemical impedance spectroscopy were conducted to evaluate the ORR performance of the catalyst. Using the small GDE setup (Supplementary Fig. 7) developed by Kellner et al.^15^, which was inspired by the design of Arenz’s group, a single frequency of 5 kHz is recommended for resistance measurement^35^. During the measurement, a forward stepwise current sweep from low to high current densities within the range of 0.1–1000 mA cm⁻² was applied, and the stabilized potential at each step was recorded. In this GDE setup, localized ohmic heating at the working electrode could potentially damage the membrane and degrade the catalyst^36^, especially at high current densities. Therefore, the maximum testing current density and the time for each step were limited to 1 A cm⁻² and 10 s for current densities ≥25 mA cm^-2^, respectively^37^.

**Fig. 2:**
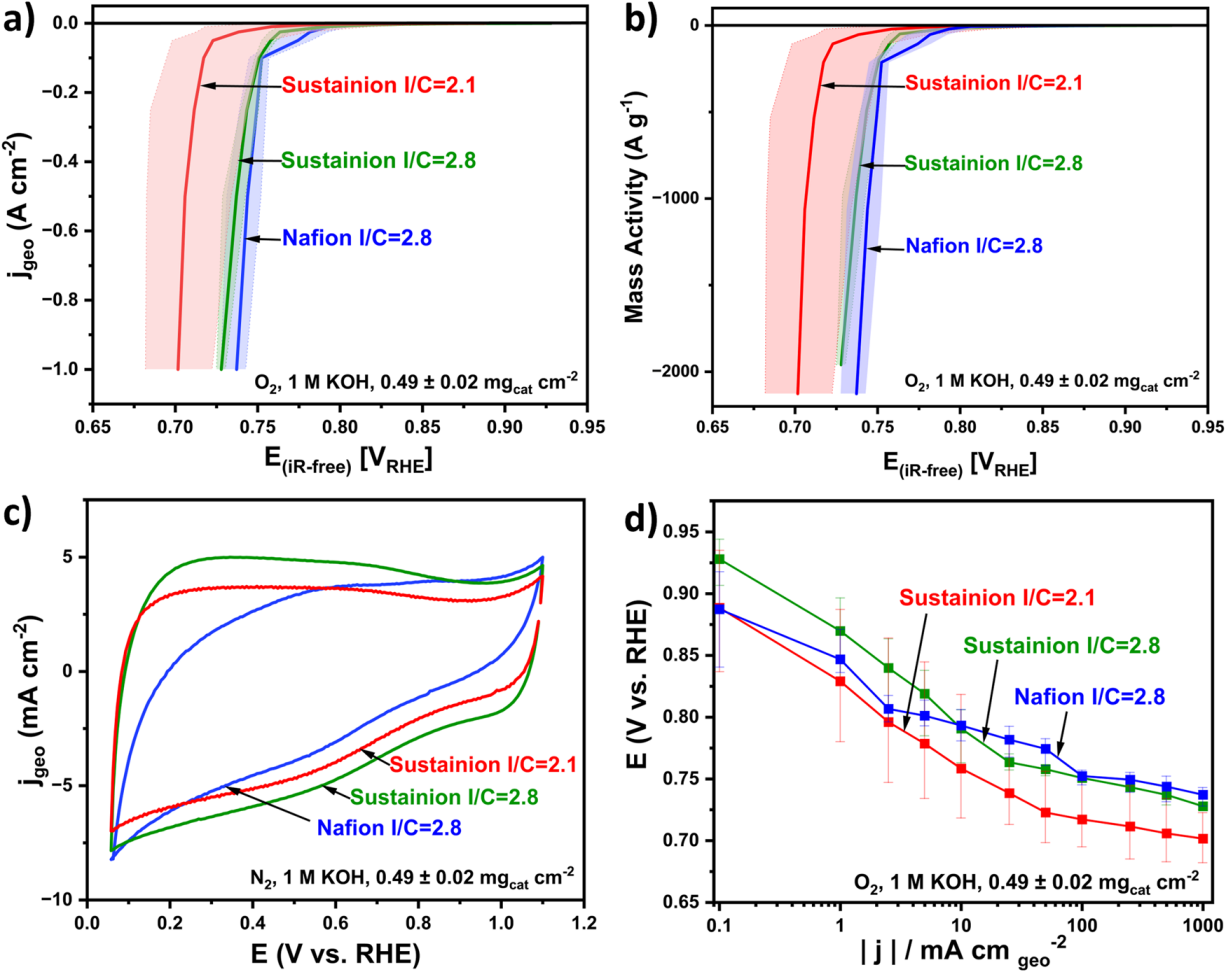
GDE results obtained from galvanostatic steps with in-situ impedance spectroscopy and CVs under N_2_ conditions. **a** Comparison of polarization curves of alkaline Fe-N-C cathodes (TAP900@Fe), with Nafion ionomer (blue, 0.47 mg_cat_ cm^−2^, *I*/*C* = 2.8), Sustainion ionomer (green, 0.51 mg_cat_ cm^−2^, *I*/*C* = 2.8) and (red, 0.47 mg_cat_ cm^−2^, *I*/*C* = 2.1). Electrodes with alkaline exchange ionomers have been activated in 1 M KOH before measurements. AEM Sustainion (X37-50 Grade 60) has been activated in 1 M KOH and used in all experiments. All experiments are tested in 1 M KOH with O_2_ at room temperature. For different electrodes, three independent measurements were carried conducted with the average value plotted and corresponding error bars. All electrochemical potential values are 100% iR-corrected. **b** Polarization curves after normalization to catalyst loading. **c** CVs comparison under nitrogen gas supply with a scan rate of 50 mV s^−1^. **d** Tafel plot for the ORR in O_2_.

As shown in Fig. 2a, it can be seen from the O_2_ polarization curve that although the functional groups of Nafion do not transfer hydroxide ions in its supplied state and could act as an ionic insulator for OH^-^^38^, the electrode with Nafion showed the best performance at high current densities, achieving 0.737 ± 0.010 V_RHE_ at 1 A cm⁻² (ref. ^39^). Hu et al. reported that as the contact time between KOH and the Nafion membrane (N212) increases, the permeation rate of hydroxide ions through the membrane increases. Such penetration of hydroxide groups through the membrane was due to the phase-separated structure arising from the hydrophobic Teflon backbone and hydrophilic sulfonic acid groups in the polymer backbone^40^. However, Hou et al. reported a low ionic conductivity for the Nafion membrane (N112), achieving 0.011 S cm^-1^ and 0.032 S cm^-1^ after being soaked in 1 M and 6 M KOH, respectively^41^. A slightly lower performance is observed with the electrode using Sustainion at an *I*/*C* ratio of 2.8, achieving 0.728 ± 0.003 V_RHE_ at 1 A cm⁻², but the difference is minor and falls within the error margins of GDE testing at high current densities. As shown in Fig. 2c, the electrode prepared employing Nafion shows the lowest double layer capacitance of 138 mF cm^-2^ (294 F g^-1^), and the Sustainion electrode with an *I*/*C* ratio of 2.8 shows the highest of 226 mF cm^-2^ (443 F g^-1^). No peaks corresponding to the Fe^III^/Fe^II^ redox (0.76 V_RHE_) were observed; however, the presence of such a peak is not a prerequisite for achieving high ORR performance^42^. Although GDE with Nafion showed the highest potential at high current density, according to the Tafel plot shown in Fig. 2d, GDEs with Sustainion ionomer (*I*/*C* = 2.8) showed higher intrinsic activity in the kinetic region and higher performance up to 10 mA cm^-2^, which could be due to the fully activated ionomer with enhanced hydroxide conductivity. The Sustainion GDEs with a higher *I*/*C* ratio of 2.8 demonstrate improved performance compared to an *I*/*C* ratio of 2.1, likely due to the increased ionomer coverage, which is essential for catalysts with high surface area and abundant porosity.

The Piperion ionomer with an *I*/*C* ratio of 2.8 has also been investigated. The average GDE performance with the Piperion ionomer (Supplementary Fig. 8), without prior ionomer activation, achieved 0.735 V_RHE_ at 1 A cm⁻². This performance was comparable to the GDE results with Sustainion and Nafion. Although no pre-activation of the GDE was performed before measurements, activation of the ionomer in the catalyst layer may have occurred during the measurement upon contact with the KOH electrolyte^43^. However, the large error bars indicated non-reproducible results and the left error bar of the polarization curve indicates an increased potential at higher current densities. This could be attributed to (1) instability of the catalyst layer when in contact with the electrolyte, leading to damage and inhomogeneity at higher current densities (>1 A cm⁻²), (2) thick catalyst layer arising from the high catalyst loading (1 ± 0.1 mg_cat_ cm⁻²) which leads to membrane deformation and (3) overcorrection of resistance at high current densities.

**Fig. 3:**
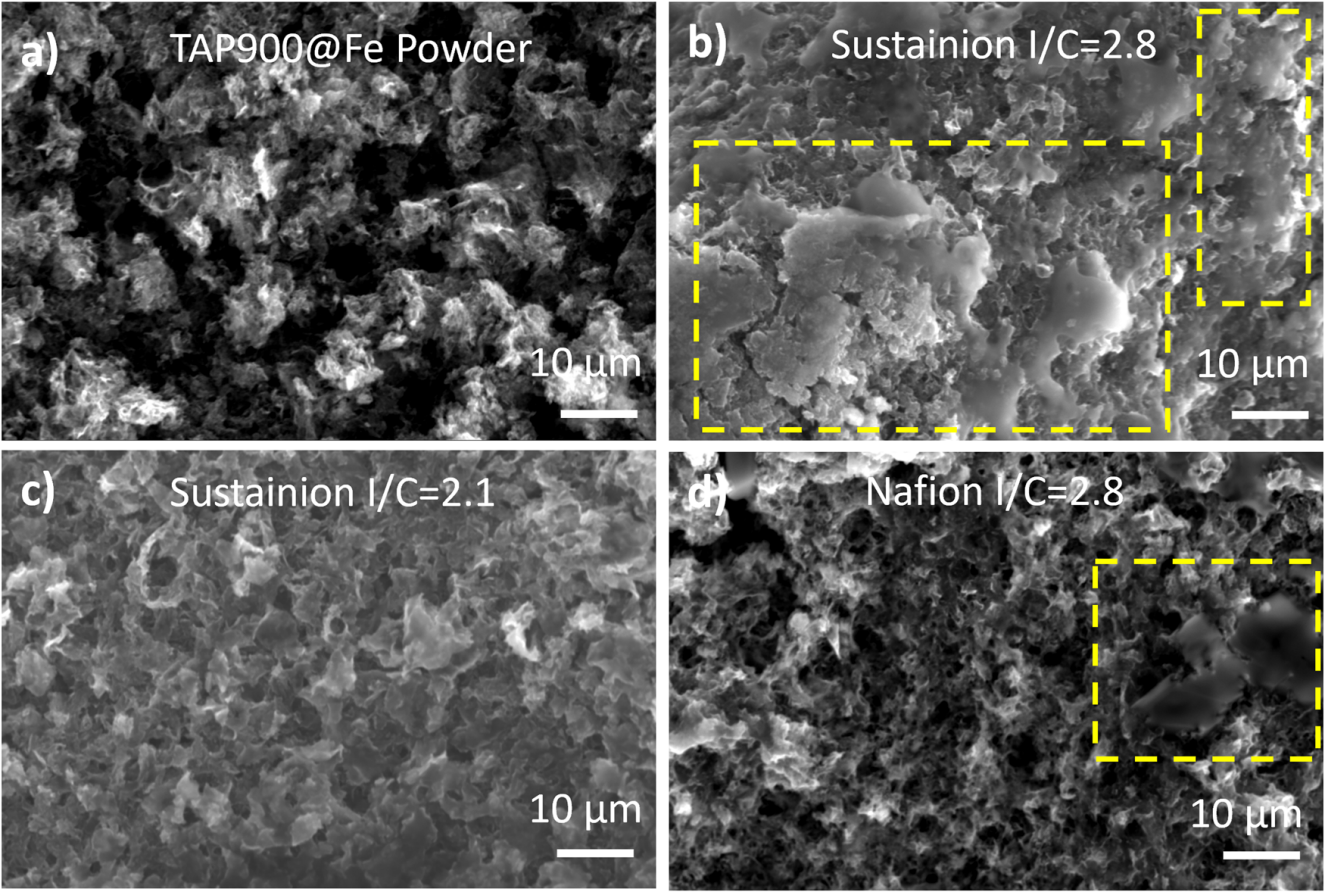
SEM images of the surface of TAP900@Fe. **a** Bare catalyst powder. Spray coated electrode before electrochemical measurement with (**b**) Sustainion *I*/*C* = 2.8, **c** Sustainion *I*/*C* = 2.1, **d** Nafion *I*/*C* = 2.8. SEM scale bars in all images represent 10 µm.

The slightly better performance of the electrode with Nafion at a current density >10 mA cm^-2^ can be explained by the catalyst layer morphology, as the quality of the GDE greatly affects the performance^44^. Jervis et al. reported that electrodes with hydroxide ionomer showed large catalyst agglomerates and poor accessibility to active sites compared to electrodes with Nafion^38^. SEM can distinguish ionomers, which have low electronic conductivity, from conductive carbon materials and can reveal changes in the electrode surface morphology as ionomer content increases. All SEM images were taken before any electrochemical measurements, and an image of the bare catalyst powders is provided for comparison (Fig. 3a). SEM showed clear ionomer aggregation with globular shape as highlighted in yellow on catalyst surfaces (*I*/*C* = 2.8), with Sustainion (Fig. 3b) forming large clusters of ionomers that cover the surface and block the pores, suggesting ionomer saturation on the catalyst surface. In contrast, with Nafion (Fig. 3d), globular features with diameters on the order of 10 µm were observed only in isolated areas. The higher ionomer coverage might lead to higher electrochemical surface area as suggested by the double layer capacitance (226 vs. 138 mF cm^-2^). However, the aggregation seen with Sustainion is expected to increase the O_2_ transport resistance and the limit O_2_ available to the active sites, leading to lower performance at high current densities. Apart from that, Nafion has been reported to show higher O_2_ permeability than anion exchange ionomers^45,46^. A lower *I*/*C* of 2.1 with Sustainion (Fig. 3c) showed clear open pores in the catalyst layer with no ionomer aggregation. These phenomena are consistent with the findings of Hyun et al.^17^, who observed aggregation with Sustainion and Pt/C, but not with Nafion, and with Pedersen et al.’s PEMFC Fe-N-C cathodes, which started to show globular features at *I*/*C* of 3.5 with Nafion^28^. We would like to note that, although the same *I*/*C* of 2.8 was applied to TAP900@Fe with Piperion ionomer (Supplementary Fig. 9c), no ionomer aggregation was seen from SEM, which could be due to different ionomer properties, interaction between ionomer and catalyst and the effect of ink formulation. The size of the ionomer aggregation could be controlled by adjusting ionomer-solvent interactions using different solvents, as suggested by an earlier study by Hyun et al., who showed that with a *meta*-Poly(terphenylene) anion exchange ionomer, the catalyst layer fabricated using dimethyl sulfoxide as the solvent resulted in a uniform structure and the highest power density while using IPA as the solvent led to pore clogging and the lowest power density in AEMFC performance^47^. In this study, IPA was used throughout for easier comparison with literature^10,14^. The Sustainion ionomer with an *I*/*C* ratio of 3.5 has also been investigated. The further increase in the *I*/*C* ratio to 3.5 resulted in enhanced ionomer coverage over the catalyst surface (Supplementary Fig. 10a, b). The scattered white spherical patterns observed represent ionomer clusters, as ionomers have lower electronic conductivity compared to carbon-based ones. At high magnification (Supplementary Fig. 10c), the catalyst layer appears almost entirely covered with ionomers. The GDE with Sustainion ionomer of *I*/*C* = 3.5 showed a lower capacitance of 123 mF cm^-2^ (238 F g^-1^) compared with *I*/*C* = 2.1 of 184 mF cm^-2^ (391 F g^-1^), which is also lower than that of Nafion (Supplementary Fig. 11a). From the polarization curve of the alkaline GDE, the resistance-corrected curve showed an increased potential with rising current densities (Supplementary Fig. 11b), which arose from the higher resistance observed in the EIS (ranging from 1 to 4 Ω which is much higher than the other GDE measurements typically showing resistance of less than 1 Ω). This behavior can be attributed to the increased mass transport resistance due to the ionomer saturating the catalyst’s pores. Furthermore, the Sustainion ionomer exhibits the highest water uptake percentage (80%) among all commercially available alkaline exchange ionomers, which could lead to significant volumetric swelling effects and thus increase resistance at the catalyst and membrane interface^24,48^.

The testing procedure proposed by the benchmarking protocol did not include the catalyst electrochemical wetting in O_2_^9^; however, Lauf et al. recently highlighted the significance of such a step in the GDE half-cell setup^43^. Their protocol, which involved alternating galvanostatic holds at 250 mA cm⁻² and 25 mA cm⁻², helped to improve ORR performance and increased Pt accessibility in the catalyst layer^43^. We also observed that the second measurement of the same electrode showed a higher performance compared with the first measurement, which could be due to a fully activated catalyst during the measurement (Supplementary Fig. 12). Pedersen et al. also showed an improvement in ORR current densities up to 50 mA cm^-2^ in their acidic Fe-N-C GDE study following an accelerated stress test protocol under Ar, which was tentatively assigned to improved wetting of the catalyst layer^36^. These protocols will be applied to our future GDE study to improve catalyst layer utilization. In summary, we have confirmed that the formulation of the catalyst layer plays a crucial role in GDE testing, even without using an anion exchange ionomer, a catalyst layer with Nafion provides a uniform electrode structure, resulting in the best alkaline GDE performance at high current density. In contrast, a lower *I*/*C* ratio of 2.1 with Sustainion may be insufficient for a highly porous catalyst to achieve good hydroxide conductivity, leading to the lowest alkaline GDE performance.

**Fig. 4:**
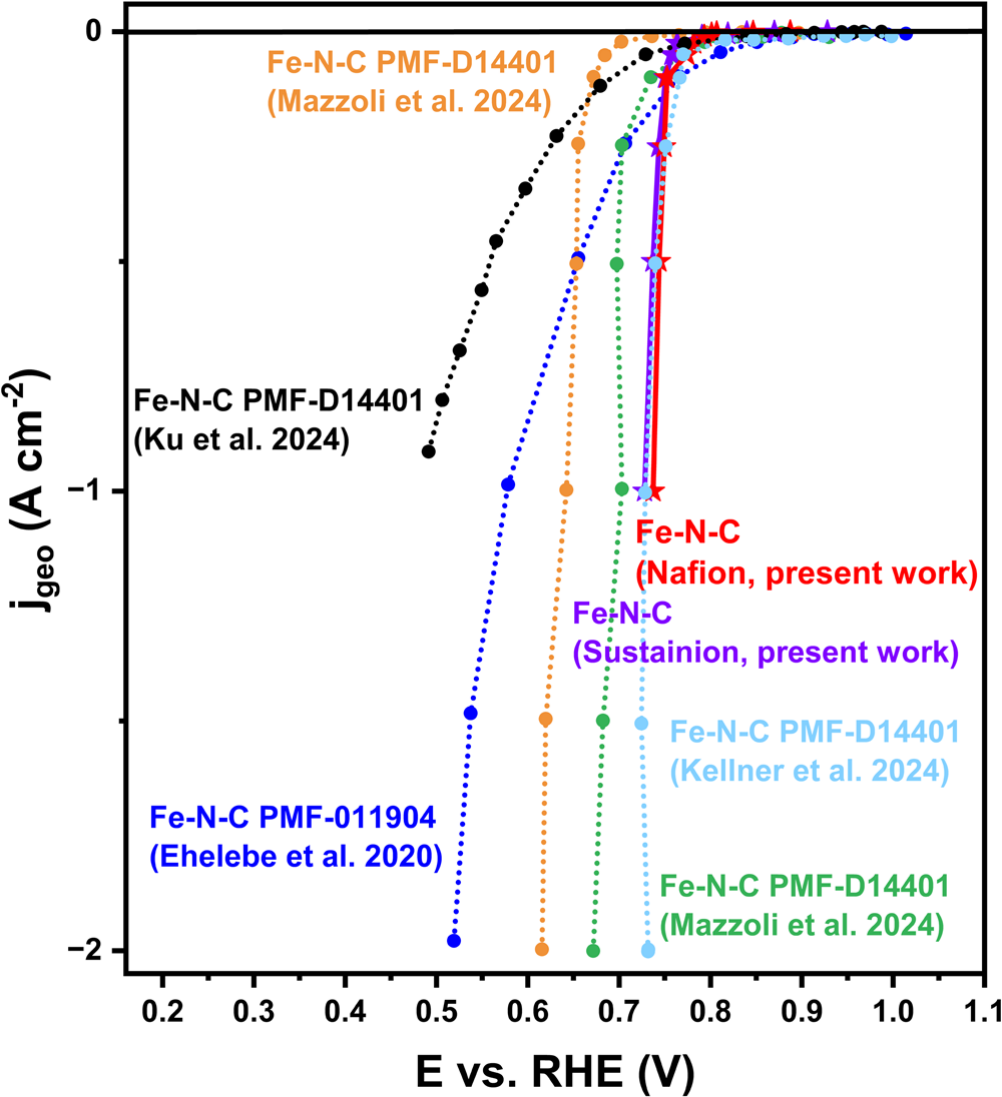
Literature comparison between present work and other Fe-N-C in GDE half-cell experiments. Present work (red stars, 0.47 mg_Fe-N-C_ cm^−2^, Nafion ionomer and (purple stars, 0.51 mg_Fe-N-C_ cm^−2^, Sustainion ionomer), Ehelebe et al.^30^: Pajarito Powder PMF-011904 (blue, 1.6 mg_Fe-N-C_ cm^−2^, Aemion ionomer)^30^, Ku et al.^14^: Pajarito Powder PMF-D14401 (black, 0.72 mg_Fe-N-C_ cm^−2^, Aemion ionomer)^14^, Kellner et al.^15^: Pajarito Powder PMF-D14401 (light blue, 1.2 mg_Fe-N-C_ cm^−2^, Piperion ionomer)^15^ and Mazzoli et al.^13^: Pajarito Powder PMF-D14401 (green, 1.29 mg_Fe-N-C_ cm^−2^, Sustainion ionomer) and Fe-N-C (orange, 1.05 mg_Fe-N-C_ cm^−2^, Sustainion ionomer)^13^.

The GDE results are compared to the performances measured in the GDE setup with Fe-N-C and with state-of-the-art AEMFC and Pt/C catalysts. From Supplementary Fig. 13, it is shown that the onset potential of the polarization curve at which the reduction reaction started and the overall performance of our catalyst is inferior to that of the Pt/C catalyst. This difference could be due to various testing parameters, such as the use of a solid ionomer developed by Varcoe and co-workers that is tailored for alkaline conditions and affects the catalyst layer and mass transport properties^49^. Despite this, our catalyst achieves a current density of 1 A cm⁻² at 0.737 V_RHE_, outperforming almost all commercial Fe-N-C Pajarito Powder in the GDE half-cell. As shown in Fig. 4, compared to the xylose-derived Fe-N-C catalyst developed by Mazzoli et al. using the same GDE setup with Sustainion ionomer and membrane, the TAP900@Fe catalyst shows an overall positive shift of 86 mV in the polarization curve up to 1 A cm^-2^ and a shift of roughly 40 mV compared to the benchmark commercial Fe-N-C (PMF-D14401, Pajarito Powder)^13^. The higher performance could be assigned to the highly accessible active sites of the catalyst even with low loading (PMF-D14401 Pajarito Powder with 1.29 mg_Fe-N-C_ cm^−2^ and Fe-N-C catalyst with 1.05 mg_Fe-N-C_ cm^−2^ compared with 0.51 mg_Fe-N-C_ cm^−2^), with a mass activity achieving 1960 A g^-1^ at 0.728 V_RHE_ (Fig. 2b). Compared with the Pajarito Powder PMF-011904 from Ehelebe et al.^30^, although TAP900@Fe with either ionomer shows lower potential before 0.1 A cm^-2^, the performance of the TAP900@Fe with Sustainion and Nafion ionomer operates at 150 and 159 mV higher at 1 A cm^-2^, respectively, demonstrating efficient mass transport of TAP900@Fe. The same commercial catalyst was later studied by Kellner et al. using Piperion ionomer. While it achieved a similar potential at 1 A cm⁻² as TAP900@Fe with Sustainion, the average (0.728 ± 0.016 V_RHE_) operated 9 mV lower than Nafion ionomer (0.737 ± 0.010 V_RHE_) at the same current density^15^.

## Conclusions

In this work, we have evaluated the alkaline oxygen reduction GDE performance of a highly porous, magnesium-templated Fe-N-C material with different ionomers and *I*/*C* ratios. We observed that a high *I*/*C* ratio is necessary for a porous Fe-N-C catalyst in alkaline environments. Through galvanostatic steps coupled with in-situ electrochemical impedance spectroscopy and SEM analysis, it is shown that a low ionomer loading of *I*/*C* = 2.1 leads to insufficient coverage of the Sustainion ionomer on the catalyst surface, and, therefore, inaccessible active sites and low performance. Conversely, at a higher ionomer loading of *I*/*C* = 2.8, the Sustainion ionomer performed the best in the kinetic region, while the Nafion ionomer produced a more homogeneous catalyst layer, resulting in the highest GDE performance at >10 mA cm^-2^, achieving 0.737 V_RHE_ at 1 A cm^2^. When comparing the GDE performance with commercial Pajarito Powder in a GDE setup from the literature, the high porosity of TAP900@Fe allows for enhanced mass transport channels, contributing to superior ORR performance in alkaline conditions. Our findings, therefore, provide new insights into the fabrication of catalyst layers with porous Fe-N-C cathodes for electrochemical technologies. Future work will focus on the optimization of the catalyst layer in the GDE setup with different anion exchange ionomers and AEMs and extend this knowledge to AEMFC testing.

## Methods

### Material synthesis

The Fe-N-C electrocatalyst is prepared as previously reported by Fe-coordination in a highly porous nitrogen-doped carbon^18^. Initially, a nitrogen-doped carbon material is prepared by pyrolysis of 2,4,6-triaminopyrimidine (TAP) and Magnesium Chloride hexahydrate (MgCl_2_·6H_2_O) at a temperature of 900 °C in an N_2_ atmosphere with 300 mL min^−1^ flow rate and 5 °C min^−1^ heating rate. After cooling down to room temperature naturally, the resulting material is washed in 2 M HCl (prepared by dilution of fuming 37% HCl, Merck) overnight to remove any MgCl_2_ or MgO species. The washed material was then filtered, rinsed with 500 mL of distilled water, dried at 80 °C in a vacuum oven for 24 h, and labeled as TAP900. Fe-N-C is then obtained via Fe-coordination within TAP900 with iron chloride (FeCl_2_) and a methanol reflux. 60 mg of TAP900 was placed in a 250 mL round-bottom flask containing 75 mL of methanol. The mixture was vigorously stirred to achieve a homogeneous dispersion. Subsequently, 75 mL of a 25 mM FeCl_2_ solution in methanol was added, and the mixture was heated under reflux at 90 °C for 24 h. Following the iron coordination step, the product was filtered, rinsed with methanol, and then washed with 0.5 M H_2_SO_4_ overnight to remove any Fe aggregates and nanoparticles. The final material is denoted as TAP900@Fe.

### Electrochemical measurements

#### RDE

Electrochemical characterization was performed using a Multi-AUTOLAB/M101 potentiostat (Metrohm) in a standard three-electrode cell setup, where a glassy carbon rod (Sigradur, HTW Hochtemperatur-Werkstoffe GmbH) served as the counter electrode, and a Hg/HgO (6 mm, als-Japan) electrode functioned as the reference. For calibration, hydrogen gas (1 bar_g_) was purged for 10 min in 0.1 M KOH (99.995% Suprapur), with a platinum rod (Metrohm) as the counter electrode, the Hg/HgO as the reference electrode, and a 3 mm Pt RDE (Metrohm) working electrode rotating at 1600 rpm throughout the process. Five cyclic voltammograms were recorded at a scan rate of 10 mV/s between −0.86 and −0.94 V _Hg/HgO_. The average value of the conversion between Hg/HgO_sat_ and RHE was obtained from forward and reverse scans starting from zero current^50^. To prepare the working electrode, an ink containing 4 mg of the catalyst, 480 µL of 18.2 MΩ cm deionized water, 480 µL of isopropanol (≥99.5% Honeywell, Fisher Scientific), and 40 µL of 5 wt% Nafion D-521 (5% w/w in water and 1-propanol, Alfa Aesar) was freshly sonicated for 30 min in a bath sonicator (132 kHz ultrasonic cleaner, VWR). 13 µL of the prepared ink was drop-cast onto a freshly polished 5 mm diameter glassy carbon RDE, which served as the working electrode. The drop-cast ink was dried at 500 rpm for 30 min in ambient air, achieving a catalyst loading of 0.26 mg_FeNC_ cm^−2^.

To start the RDE measurements, cyclic voltammograms (CVs) were recorded following a 15-min N₂ purge (99.9998%, Ultrapure Plus) at 50 mV s⁻¹ and 0 rpm over a potential range of 0.2 to 1.1 V_RHE_ to precondition the catalyst. Subsequently, voltammograms were obtained at 10 mV s⁻¹ and 1600 rpm over the same potential range under N₂ and O_2_ saturation (measurements at the third cycle displayed). Capacitance was corrected by subtracting the N_2_-saturated measurements at 1600 rpm from the O_2_-saturated results. Electrochemical impedance measurements were conducted by taking the first intercept on the real impedance axis of the Nyquist plot at 0.85 V and 1600 rpm, spanning frequencies from 10^5^ to 10^-1^ Hz, to determine the ohmic drop for each measurement.

The kinetic current densities (${j}_{{kin}}$) were calculated at 0.9 or 0.8 V_RHE_ using the geometric disk current density (${j}_{d}$) at 0.9 or 0.8 V_RHE_ and the limiting current density (${j}_{{\mathrm{lim}}}$) at 0.3 V_RHE_ following Eq. 1:

1 $${j}_{{kin}}=\frac{{j}_{d}\cdot {j}_{{\mathrm{lim}}}}{{j}_{d}-{j}_{{\mathrm{lim}}}}$$

The mass activity (${MA}$) is calculated with the following Eq. 2:

2 $${MA}=\,\frac{{j}_{{kin}}}{{Catalyst\; Loading}}$$

#### GDE

Electrochemical characterization was performed in a modified small gas-diffusion-electrode (GDE) cell^35^ using a potentiostat PGSTAT204 with an FRA32M Module (Metrohm), in combination with a BOOSTER10A. Catalyst inks were obtained by ultrasonic mixing of TAP900@Fe, Nafion D-521(5% w/w in water and 1-propanol, Alfa Aesar), or Piperion (5 wt% in ethanol, Fuel Cell Store), or Sustainion XA-9 (5 wt% in ethanol, Dioxide Materials) with IPA for 30 min in an ice-cooled bath sonicator (132 kHz ultrasonic cleaner, VWR), followed by overnight stirring. The catalyst ink was then loaded into the Sonotek ultrasonic sprayer (Sono-Tek ExactaCoat, Sono-Tek Corporation) and sprayed onto the gas-diffusion layer (GDL) (H23-C8, Freudenberg). The ultrasonic nozzle was operated at 120 kHz with an ultrasonic power of 1.4 W. The ink composition for the GDE had an *I*/*C* of 2.1, 2.8, or 3.5, with a catalyst content of 1.5 mg_Fe-N-C_ mL^-1^. For a typical ink formulation with *I*/*C* = 2.1, 30 mg of TAP900@Fe and 1.37 mL of Nafion, or 1.52 mL of Piperion, or 1.59 mL of Sustainion were used to form a 20 mL solution, with the remainder being IPA. The ink was then automatically sprayed at 0.5 mL min^-1^ with an offset serpentine spray pattern onto the hydrophobic side of a 5 cm² GDL (H23-C8, Freudenberg), while heated at 60 °C on a vacuum plate (air shaping at 0.8 to 2.5 psi). The gas-diffusion layer was weighed before and after catalyst spraying to determine the cathode catalyst loading (0.49 ± 0.02 mg_cat_ cm^-2^).

Prior to electrochemical measurements, the anion exchange ionomer was activated by submerging the GDE in 1 M KOH for 1 h, followed by a second 1-h wash in fresh 1 M KOH to convert the Piperion from its bicarbonate form or Sustainion from its chloride form to the desired hydroxide form. The anion exchange membrane (AEM) was activated by soaking it in 1 M KOH electrolyte for 24 h before the measurement to achieve good hydroxyl transport.

For the working electrode preparation, a piece of electrode (Ø = 3 mm) was punched out of the GDE and inserted into a punched-out hole (Ø = 3 mm) in the gas-diffusion layer (GDL) (H23C8, Freudenberg). The AEM Sustainion (X37-50 Grade 60, Fuel Cell Store) membrane (Ø = 20 mm) was positioned between the upper cell body and the H23C8 GDL, with its hydrophobic coating facing the AEM and the piece of GDE catalyst layer facing the membrane, positioned below the hole of the upper cell body. The lower cell body, with a flow field facing towards the H23C8 GDL, was connected to a pre-bubbler (for humidification) and a gas flow meter (Bronkhorst) with a flow rate of 250 mL min^-1^. The counter electrode was a platinum coil. A homemade Pt wire in hydrogen reversible hydrogen electrode (RHE) was used as the reference electrode and prepared before each measurement. The compartment of the RHE was in contact with the AEM via a Luggin capillary. The upper cell compartment of the GDE was filled with 15 mL of 1 M aqueous KOH (99.995% Suprapur). The measurements were conducted at room temperature, and protocols for the GDE half-cell are described in detail in the Supplementary Methods Electrochemistry section.

#### Material characterization

Material characterization details are provided in the Supplementary Methods Characterization section.

## Supplementary information

Supplementary Information

## Data Availability

The data that supports the findings of this study are available on the Zenodo research data repository at 10.5281/zenodo.14393678.
