# Supplementary material for: Impact of ionomers on porous Fe-N-C catalysts for alkaline oxygen reduction in gas diffusion electrodes
Source: Commun Chem. 2025 Jan 31;8:27. doi: 10.1038/s42004-025-01422-4 (PMC11785744; doi:10.1038/s42004-025-01422-4)
Supplement: Supplementary file 1 — Supplementary Information [file 42004_2025_1422_MOESM1_ESM.pdf]

Supporting Information:

Impact of ionomers on porous Fe-N-C catalysts for alkaline oxygen reduction in gas diffusion electrodes

Jinjie Zhu[a], Angus Pedersen[ab], Simon Kellner[a], Robert D. Hunter[a], Jesús Barrio[a]

[a]*Department of Chemical Engineering, Imperial College London, London SW7 2AZ, UK.*

[b]*Department of Materials, Royal School of Mines, Imperial College London, London SW7 2AZ, UK.*

Supplementary Methods

Characterization

XPS analysis was conducted using a Thermo Fisher K-Alpha XPS system, and the spectra were analyzed with Avantage software. All spectra were calibrated relative to the carbon C1s peak at 284.8 eV to correct for charging effects. XRD patterns (0.016° scan step size) were obtained using a PANalytical X'PERT PRO powder X-ray diffractometer with a Cu Kα source operating at 45 kV and 30 mA. N_2 sorption isotherms were conducted at -196°C in the pressure range of 10^{-5} to 0.99 using a Micromeritics 3Flex system. The samples (10 to 15 mg) were degassed for 16 h at 200 °C. The BET specific surface area was obtained using BET Surface Identification software[1] by analyzing the adsorption isotherm in the relative pressure range of 0.995, as determined by the Rouquerol method.[2] The pore size distribution was obtained from the adsorption isotherm using the heterogeneous surface carbon 2D-non-local density functional theory (NLDFT) method, which incorporates more realistic energetic heterogeneity and geometrical corrugation of the carbon surface and avoids artifacts.[3] The SEM analysis was conducted employing Quanta FEG 250 at 20 kV and 1000 magnification.

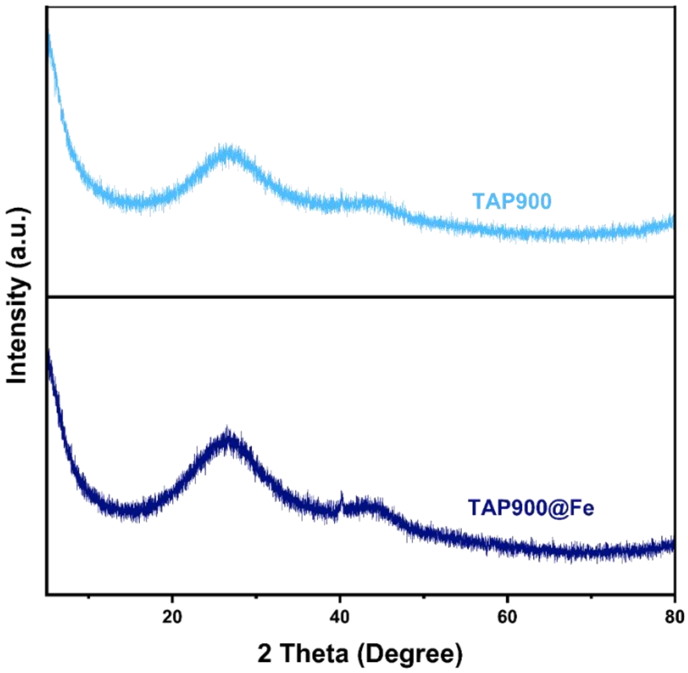

Supplementary Figure 1. XRD pattern comparison between TAP900 and TAP900@Fe.

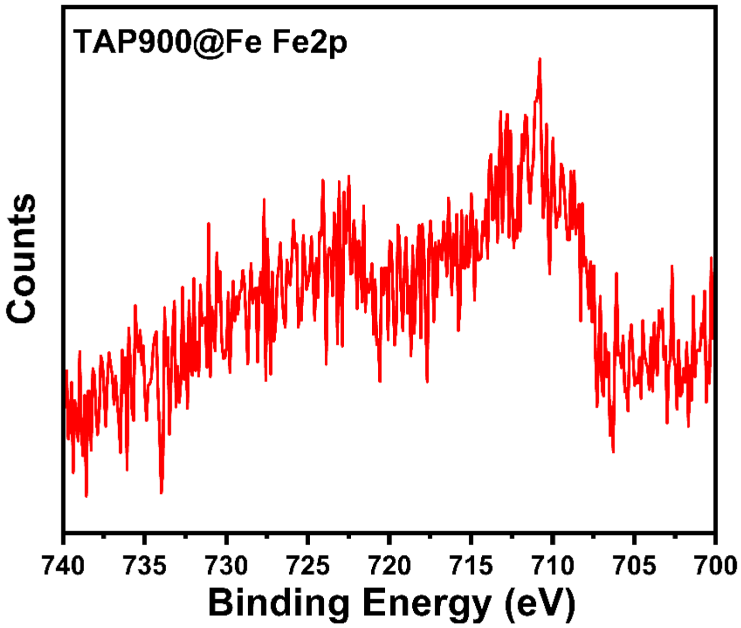

Supplementary Figure 2. Fe2p XPS spectra of TAP900@Fe.

Supplementary Table 1. Chemical composition in atomic and weight percentage of TAP900@Fe obtained by XPS.

Sample	C1s		O1s		N1s		Mg1s		Fe2p	
	At. %	Wt. %	At. %	Wt. %	At. %	Wt. %	At. %	Wt. %	At. %	Wt. %
TAP900@Fe	84.01	78.35	7.41	9.21	7.32	7.96	0.4	0.76	0.86	3.72

Supplementary Table 2. Comparison of TAP900@Fe with other Mg-derived Fe-N-C catalysts in terms of Mg to precursor mass ratio, BET specific surface area, total pore volume, and meso- and macropore volume.

Sample	Mg source	Mg to precursor mass ratio	Specific surface area ($m^2\ g^{-1}$)	Total pore volume ($cm^3\ g^{-1}$)	Mesopore and Macropore volume ($cm^3\ g^{-1}$)	Ref
ADNC-Fe	$MgCl_2 \cdot 6H_2O$	9.6	2500	-	-	[4]
FeNC(Mg)	$Mg(CH_3COO)_2 \cdot 4H_2O$	0.045	483	0.61	0.47	[5]
FeNC-MgOAc	$Mg(CH_3COO)_2 \cdot 4H_2O$	0.088	720	1.08	0.91	[6]
NC-800	$MgCl_2 \cdot 6H_2O$	10	2680	3.42	2.94	[7]
Ade-Mg-5-900	$MgCl_2 \cdot 6H_2O$	5	2780	2.83	-	[8]
C_2N 900	$MgCl_2 \cdot 6H_2O$	8	2111	-	-	[9]
PENG	$Mg(CH_3COO)_2 \cdot 4H_2O$	0.6	741	1.37	-	[10]
Fe(N/C 900)	$MgCO_3 \cdot 2H_2O$	0.73	1614	1.62	1.42	[11]
FeNC-LT2	$Mg(NO_3)_2 \cdot 6H_2O$	1	1223	-	-	[12]
$Fe-N_4$-PC-2	$Mg(OH)_2$	2.5	1137	-	-	[13]
$Fe–N_4$/HPC	$MgCl_2 \cdot 6H_2O$	1	664	-	-	[14]
NCMS-Fe	$Mg_5(CO_3)_4(OH)_2 \cdot 4H_2O$	1	623	-	-	[15]
TAP900@Fe (this work)	$MgCl_2 \cdot 6H_2O$	8	3615	3.11	2.87	-

Supplementary Note 1:

Although TAP900@Fe exhibited the highest specific surface area of 3293 $m^2\ g^{-1}$ among these materials, its active site density was not the highest, reaching 2.54 × 10^{19} sites g^{-1} (Supplementary Table 3). This is lower than the recent materials derived from ZIF-8, which achieved 4.67 × 10^{19} from Mehmood *et al.* [16] and 6 × 10^{19} sites g^{-1} from Jiao *et al.*[17], and the material developed by Tolosana *et al.* [18] who achieved 2.78 × 10^{19} sites g^{-1}. Apart from that, the number of active sites per unit area derived from the high specific surface area was 0.77 × 10^{16} sites m^{-2}, which is only higher than the PAJ material from Primbs *et al.*[19], who reported 0.42 × 10^{16} sites m^{-2}. The remarkably high site density reported by Chen *et al.* [20] and Wang *et al.* [21]showed low Fe site density based on ICP-MS measurements, resulting in Fe utilization exceeding 100%. This suggests the presence of artifacts in the measurement process.

Supplementary Table 3. Comparison of TAP900@Fe with other Fe-N-C catalysts in terms of the number of active sites per gram (derived from in-situ nitrite stripping), BET specific surface area, and the corresponding number of active sites per unit area.

Samples	In-situ Nitrite stripping ($\cdot 10^{19}$ sites g^{-1})	S_{BET} ($m^2\ g^{-1}$)	Number of active sites per area ($\cdot 10^{16}$ sites m^{-2})	Ref
Im-FeNC-1HT	2.78	650	4.3	18
sur-FeN4-HPC	34.7	1263	27	20
NC-NaCl	26.3	1911	14	21
CNRS	1.44	840	1.7	22
ICL	0.86	463	1.9	23
PAJ	0.25	593	0.42	19
UNM	0.63	763	0.83	19
FeNC-CVD-750	6	1593	3.8	17
Fe–$NC^{\Delta\text{-DCDA}}$	4.67	1155	4.04	16
TAP900@Fe	2.54	3293	0.77	24
TPI@Z8(SiO2)-650-C	1.33	1648	0.81	25

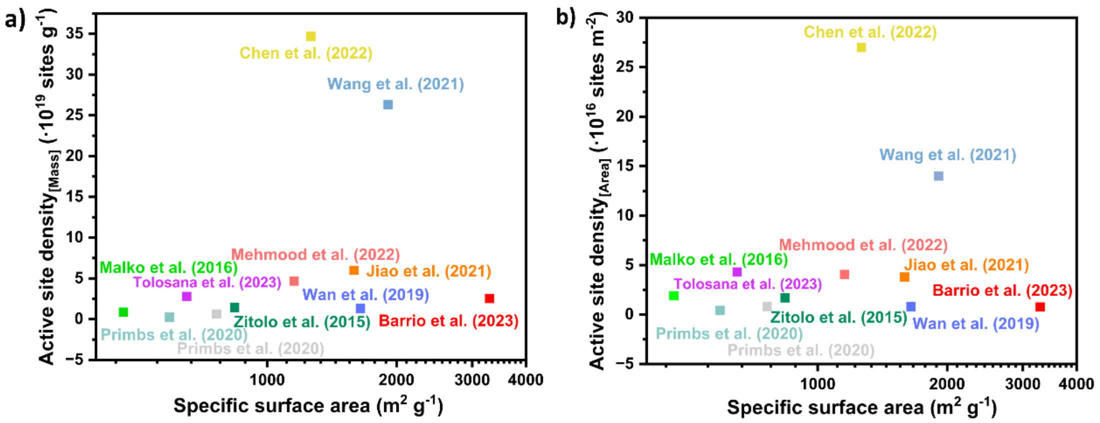

Supplementary Figure 3. Active site density comparison of TAP900@Fe with other Fe-N-C catalysts. a) number of active sites per gram (derived from in-situ nitrite stripping) and b) the number of active sites per unit area versus BET specific surface area.

Electrochemistry

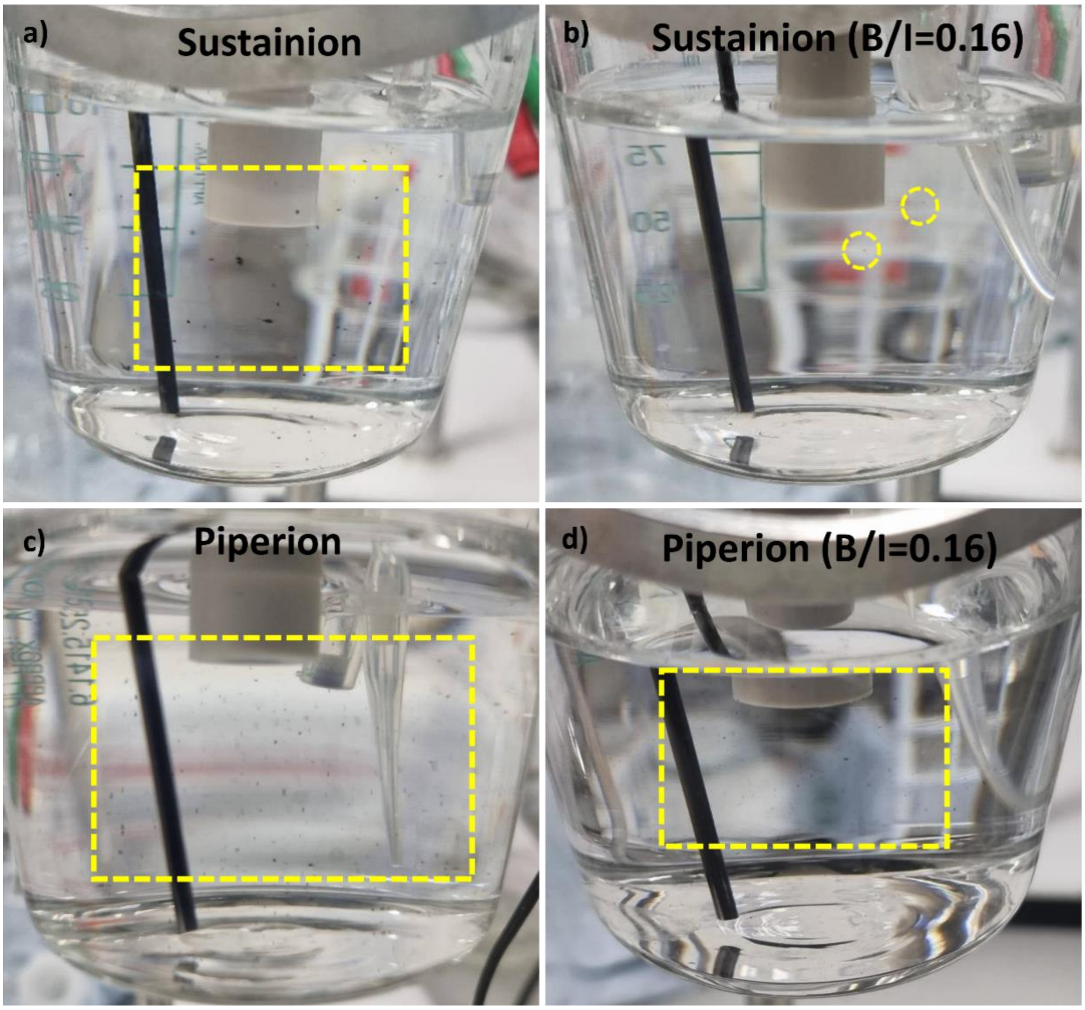

Supplementary Figure 4. Evidence of catalyst detachment with different ionomers. RDE prepared with a) Sustaionion ionomer only b) Sustainion and Nafion (B/I= 0.16) c) Piperion ionomer only and d) Piperion and Nafion (B/I= 0.16) under rotation at 1600 rpm in 0.1 M KOH.

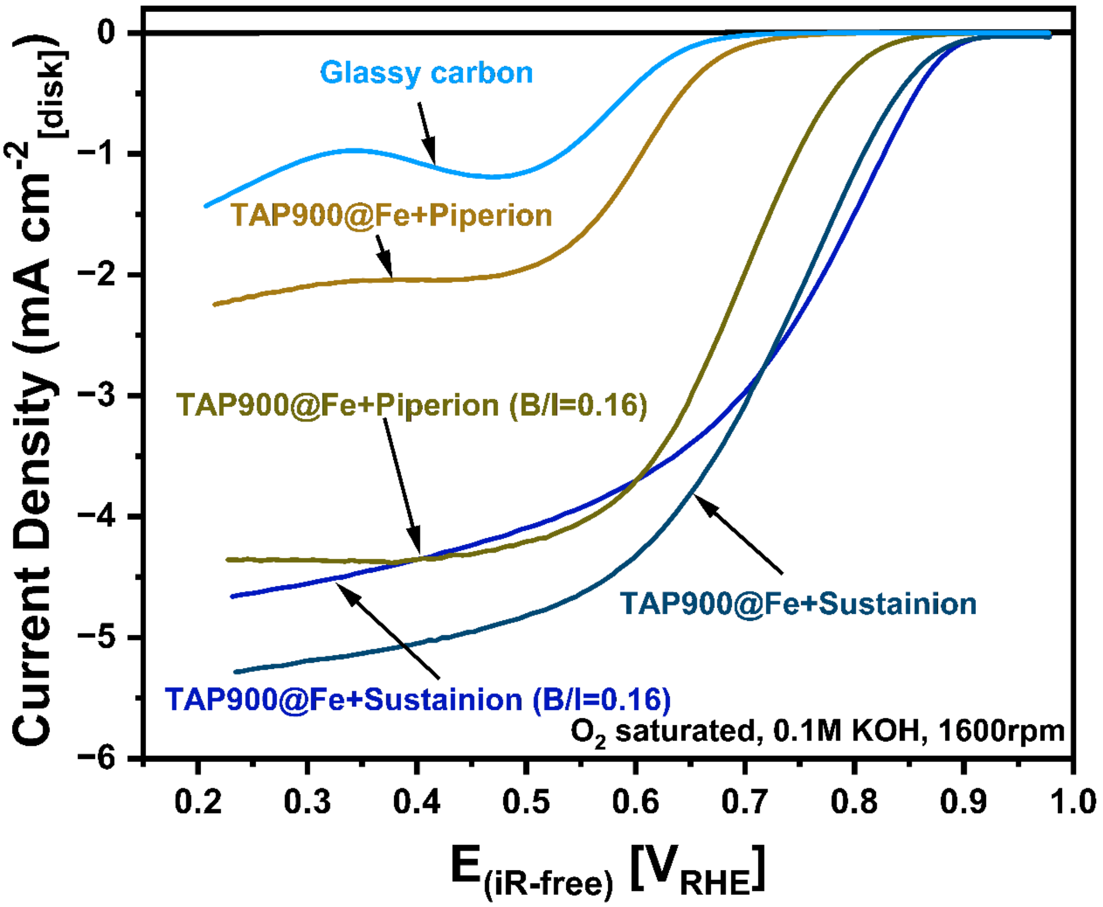

Supplementary Figure 5. The cyclic voltammogram comparison of glassy carbon, TAP900@Fe with Sustainion ionomer only, TAP900@Fe with Sustainion and Nafion (B/I = 0.16), TAP900@Fe with Piperion ionomer only, and TAP900@Fe with Piperion and Nafion (B/I = 0.16). These measurements were obtained using a rotating disk electrode (RDE) in 0.1 M KOH (Suprapur) at room temperature, with a rotation speed of 1600 rpm recorded at 10 mV s^{-1} obtained with capacitance and iR correction. The catalyst loading was 0.26 mg_{FeNC} cm^{-2}.

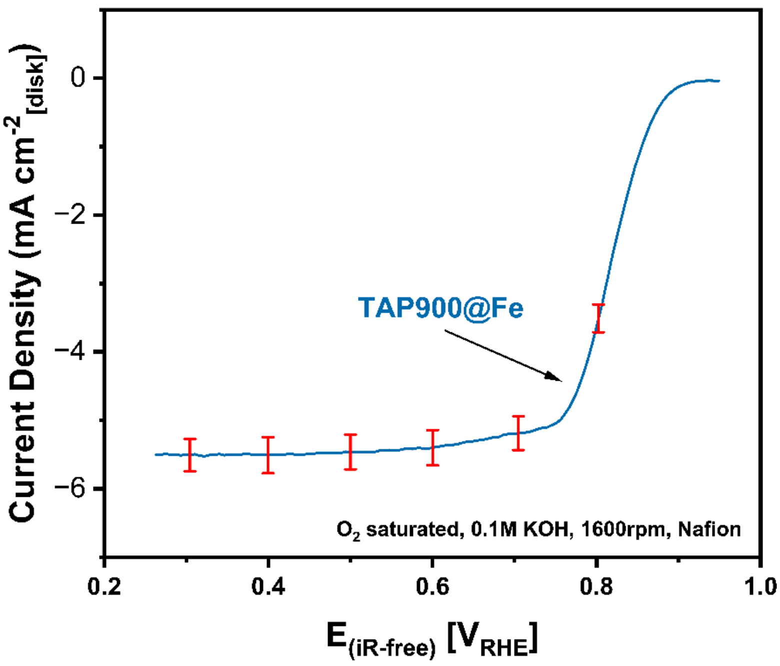

Supplementary Figure 6. Cyclic voltammogram obtained from RDE in 0.1 M KOH (Suprapur) at room temperature with a rotation speed of 1600 rpm recorded at 10 mV s^{-1} obtained with capacitance and iR correction. The catalyst loading was 0.26 mg_{FeNC} cm^{-2}. For TAP 900@Fe, two independent measurements were carried out with the average value plotted and error bars correspond to the standard deviation at the given potentials.

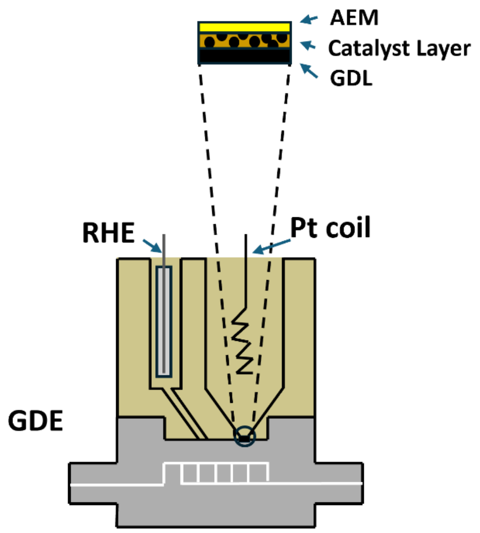

Supplementary Figure 7. Schematic of the GDE setup used throughout the study.

The GDE testing protocol was applied based on the benchmarked GDE measurement protocol with slight modification.[26] To prepare for the measurements, the catalyst layer was conditioned by subjecting it to potential cycling between 0.06 and 1.10 V vs. RHE in nitrogen atmosphere until a stable cyclic voltammogram was obtained. A cyclic voltammetry of 20 cycles at a scan rate of 500 mV s^{-1} and 10 cycles at a scan rate of 50 mV s^{-1} was used to precondition the catalyst. Prior to commencing O_2 reduction measurements, the working electrode (WE) was purged with a flow rate of 250 ml min^{-1} of humidified O_2 for a duration of 15 minutes. Subsequently, galvanostatic steps coupled with Electrochemical Impedance Spectroscopy (EIS) were employed. Current steps were applied, with corresponding hold times, including -0.1 mA cm^{-2} (90 seconds), -1/-2.5 mA cm^{-2} and -5/-10 mA cm^{-2} (30 seconds), and -25/-50/-100/-250/-500/-1000 mA cm^{-2} (10 seconds). At each step, an EIS, 10 – 0.1 kHz with 10 points per decade was recorded for 100% post-correction with an applied amplitude being 10% of the applied current (min. 5 mA cm^{-2}) [26] and a single frequency of 5 kHz used for resistance measurement. [27] An example of how to obtain the ORR polarization curve is detailed in Supplementary Table 4. The sample is TAP900@Fe with Sustainion ionomer with I/C=2.8. The current applied is corresponding to the current densities mentioned earlier with a working electrode area of 0.0707cm^{-2}.

Supplementary Table 4. Example of obtaining the oxygen reduction polarization curve with galvanostatic steps coupled with electrochemical impedance spectroscopy.

**Current (A)**	**Potential (V)**	**Resistance (Ω)**	**iR-free potential (V)**
-7.07E-6	0.907	0.65	0.907
-7.07E-5	0.826	0.62	0.826
-1.77E-4	0.796	0.36	0.796
-3.53E-4	0.785	0.35	0.785
-7.07E-4	0.763	0.36	0.763
-0.00177	0.756	0.39	0.757
-0.00353	0.751	0.39	0.752
-0.00707	0.746	0.38	0.748
-0.0177	0.733	0.38	0.740
-0.03535	0.726	0.38	0.740
-0.0707	0.704	0.38	0.730

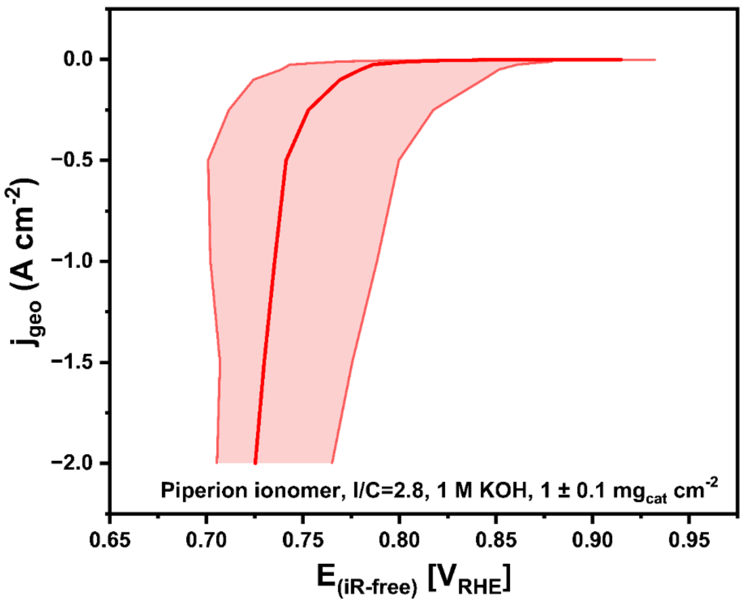

Supplementary Figure 8. Polarization curves of alkaline Fe-N-C cathodes (TAP900@Fe) obtained from galvanostatic steps with in-situ impedance spectroscopy, with Piperion ionomer and a catalyst loading of 1 ± 0.1 mg_{cat} cm^{-2}. No GDEs are activated in 1M KOH. Five independent measurements were carried conducted with the average value plotted and corresponding error bars.

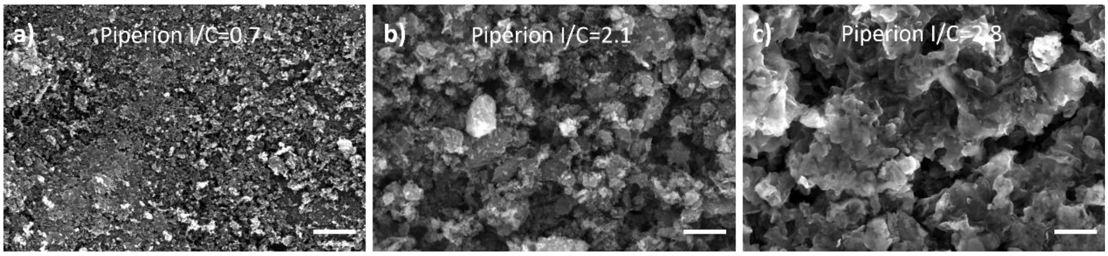

Supplementary Figure 9. SEM images of the surface of TAP900@Fe layer with Piperion ionomer. a) I/C=0.7 b) I/C=2.1 c) I/C=2.8. SEM scale bars in all images represent 10 µm.

The double layer capacitance, C_{dl}, was calculated based on integrating the cyclic voltammogram between 0.2 and 0.4 V, based on the following equation:

$$C_{dl} = \frac{1}{\Delta E} \int_{0.2\ V}^{0.4\ V} \frac{i}{v}\, d\, E \qquad \text{(Equation S1)}$$

Where v is the scan rate of 50 mV s^{-1}, i is the current in mA and ΔE is the window of integration which is 0.2 V. C_{dl} was assumed to be independent of the potential. Specific capacitance with unit A g^{-1} can be calculated by C_{dl} divided by the mass of the GDE.

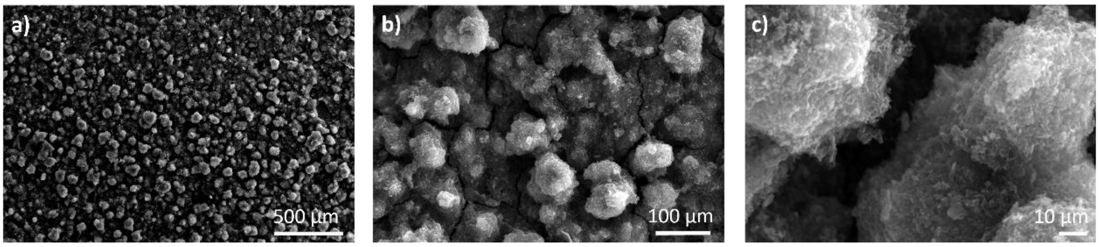

Supplementary Figure 10. SEM images of the surface of TAP900@Fe layer with Sustainion ionomer. a)× 50 b)×500 c)×1000 magnification.

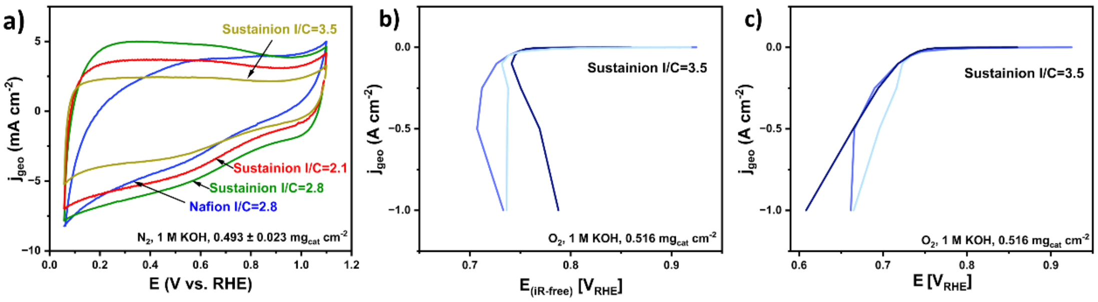

Supplementary Figure 11. CV and polarization curves results of TAP900@Fe with Sustainion ionomer of I/C = 3.5. a) CVs comparison of all GDEs under nitrogen gas supply with a scan rate of 50 mV s^{-1}. Comparison of Polarization curves of alkaline Fe-N-C cathodes (TAP900@Fe) obtained from galvanostatic steps with in-situ impedance spectroscopy b) after and c) before resistance correction between three independent measurements, with Sustainion ionomer of I/C = 3.5 and a catalyst loading of 0.516 mg_{cat} cm^{-2}. All GDEs have been activated in 1M KOH.

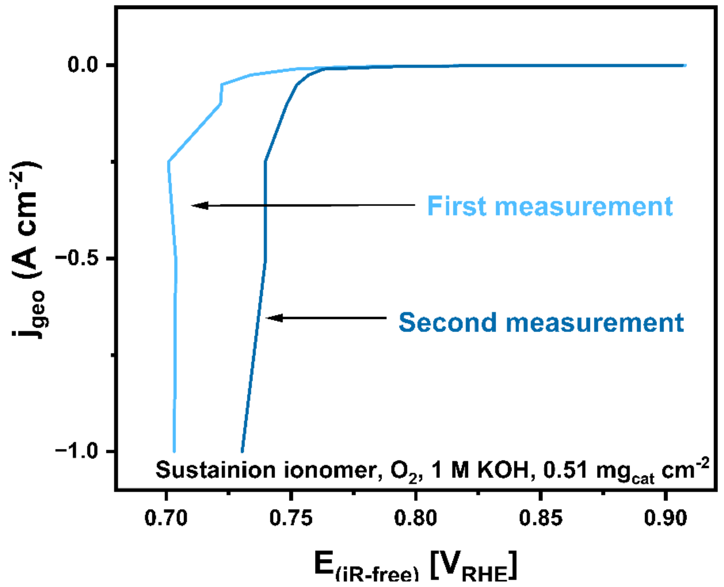

Supplementary Figure 12. Comparison of Polarization curves of alkaline Fe-N-C cathodes (TAP900@Fe) obtained from galvanostatic steps with in-situ impedance spectroscopy between two measurements. Sustainion ionomer is used with a catalyst loading of 0.51 mg_{cat} cm^{-2}. All GDEs have been activated in 1M KOH.

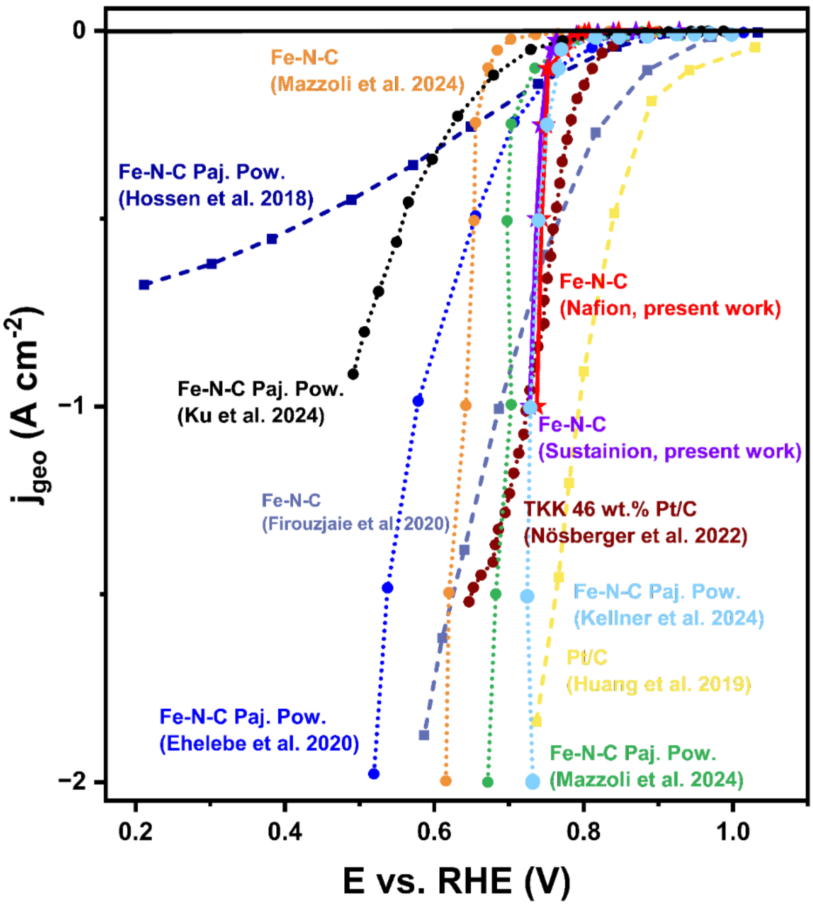

Supplementary Figure 13. Literature comparison of present work in GDE half-cell with benchmark results of AEMFC and GDE half-cell experiments. Present work (red stars, 0.47 $mg_{Fe\text{-}N\text{-}C}$ cm^{-2}, Nafion ionomer and purple stars, 0.51 $mg_{Fe\text{-}N\text{-}C}$ cm^{-2}, Sustainion ionomer). AEMFC experiments are shown in square. Hossen *et al.* 2018: Fe-N-C catalysts (dark blue, Fe-N-C cathode: 3.5 $mg_{Fe\text{-}N\text{-}C}$ cm^{-2}, Pt/C anode: 0.2 mg_{Pt} cm^{-2}, Tokuyama AS4 ionomer)[28], Firouzjaie *et al.* 2020: Fe-N-C catalysts (grey, Fe-N-C cathode: 0.9 $mg_{Fe\text{-}N\text{-}C}$ cm^{-2}, PtRu/C anode: 0.6 mg_{PtRu} cm^{-2}, ETFE solid powder ionomer[29])[30] and Huang *et al.* 2019: Pt/C catalyst (yellow, Pt/C cathode: 0.56 mg_{Pt} cm^{-2}, PtRu/C anode: 0.986 mg_{PtRu} cm^{-2}, ETFE solid ionomer[31])[32] GDE half-cell experiments are shown in circle. Nösberger *et al.* 2022: Pt/C catalyst (brown, 208 ug_{Pt} cm^{-2}, Nafion ionomer)[33], Ehelebe *et al.* 2020: Pajarito Powder (light blue, 1.6 $mg_{Fe\text{-}N\text{-}C}$ cm^{-2}, Aemion ionomer)[34], Ku *et al.* 2024: Pajarito Powder (black, 0.72 $mg_{Fe\text{-}N\text{-}C}$ cm^{-2}, Aemion ionomer)[35], Kellner *et al.* 2024: Pajarito Powder PMFD14401 (light blue, 1.2 $mg_{Fe\text{-}N\text{-}C}$ cm^{-2}, Piperion ionomer)[36] and Mazzoli *et al.* 2024: Pajarito Powder (orange, 1.29 $mg_{Fe\text{-}N\text{-}C}$ cm^{-2}, Sustainion ionomer)[37] and Fe-N-C catalysts (green, 1.05 $mg_{Fe\text{-}N\text{-}C}$ cm^{-2}, Sustainion ionomer)[37].

Supplementary Reference

1 Osterrieth, J. W. M. *et al.* How Reproducible are Surface Areas Calculated from the BET Equation? *Adv Mater* **34**, e2201502 (2022).

2 Rouquerol, J. *et al.* *Adsorption by powders and porous solids: principles, methodology and applications*. (Academic press, 2013).

3 Jagiello, J. & Olivier, J. P. 2D-NLDFT adsorption models for carbon slit-shaped pores with surface energetical heterogeneity and geometrical corrugation. *Carbon* **55**, 70-80 (2013).

4 Li, J. C. *et al.* Ionothermal-Transformation Strategy to Synthesize Hierarchically Tubular Porous Single-Iron-Atom Catalysts for High-Performance Zinc-Air Batteries. *ACS Appl Mater Interfaces* **13**, 58576-58584 (2021).

5 Sajjad, U. *et al.* Lignin-Derived Precious Metal-Free Electrocatalysts for Anion-Exchange Membrane Fuel Cell Application. *ACS Catal.* **14**, 9224-9234 (2024).

6 Kisand, K. *et al.* Templated Nitrogen-, Iron-, and Cobalt-Doped Mesoporous Nanocarbon Derived from an Alkylresorcinol Mixture for Anion-Exchange Membrane Fuel Cell Application. *ACS Catal.* **12**, 14050-14061 (2022).

7 Mehmood, A. *et al.* Facile Metal Coordination of Active Site Imprinted Nitrogen Doped Carbons for the Conservative Preparation of Non - Noble Metal Oxygen Reduction Electrocatalysts. *Adv. Energy Mater.* **8**, 1701771 (2017).

8 Pampel, J., Mehmood, A., Antonietti, M. & Fellinger, T. P. Ionothermal template transformations for preparation of tubular porous nitrogen doped carbons. *Mater. Horiz.* **4**, 493-501 (2017).

9 Barrio, J. *et al.* Metal coordination in C(2)N-like materials towards dual atom catalysts for oxygen reduction. *J. Mater. Chem. A* **10**, 6023-6030 (2022).

10 Qin, L. *et al.* Facile synthesis of porous nitrogen-doped holey graphene as an efficient metal-free catalyst for the oxygen reduction reaction. *Nano Res.* **10**, 305-319 (2016).

11 Koyuturk, B. *et al.* A simple decagram-scale synthesis of an atomically dispersed, hierarchically porous Fe–N–C catalyst for acidic ORR. *J. Mater. Chem. A* **10**, 19859-19867 (2022).

12 Kisand, K. *et al.* Hierarchically Porous Fe-N-C Single-Atom Catalysts via Ionothermal Synthesis for Oxygen Reduction Reaction. *ChemSusChem*, e202401332 (2024).

13 Wang, J. *et al.* Facile Synthesis of Atomic Fe-N-C Materials and Dual Roles Investigation of Fe-N(4) Sites in Fenton-Like Reactions. *Adv. Sci.* **8**, e2101824 (2021).

14 Yu, J. *et al.* Preparation of a high-yield Fe−N4 site-containing hierarchical porous carbon eletrocatalyst for effective oxygen reduction and zinc-air battery: The effect of dual protection-exposure mechanism. *Int. J. Hydrogen Energy* **53**, 325-334 (2024).

15 Cheng, P. *et al.* Fe/N co-doped carbon microspheres as a high performance electrocatalyst for the oxygen reduction reaction. *RSC Adv.* **5**, 107389-107395 (2015).

16 Mehmood, A. *et al.* High loading of single atomic iron sites in Fe–NC oxygen reduction catalysts for proton exchange membrane fuel cells. *Nat. Catal.* **5**, 311-323 (2022).

17 Jiao, L. *et al.* Chemical vapour deposition of Fe-N-C oxygen reduction catalysts with full utilization of dense Fe-N(4) sites. *Nat. Mater.* **20**, 1385-1391 (2021).

18 Tolosana-Moranchel, Á. *et al.* Metal-doped imine frameworks for the oxygen reduction reaction in acidic media. *J. Power Sources* **578**, 233223 (2023).

19 Primbs, M. *et al.* Establishing reactivity descriptors for platinum group metal (PGM)-free Fe–N–C catalysts for PEM fuel cells. *Energy Environ. Sci.* **13**, 2480-2500 (2020).

20 Chen, G. *et al.* Highly accessible and dense surface single metal FeN4 active sites for promoting the oxygen reduction reaction. *Energy Environ. Sci.* **15**, 2619-2628 (2022).

21 Wang, Q. *et al.* Molten NaCl‐Assisted Synthesis of Porous Fe‐N‐C Electrocatalysts with a High Density of Catalytically Accessible FeN4 Active Sites and Outstanding Oxygen Reduction Reaction Performance. *Adv. Energy Mater.* **11**, 2100219 (2021).

22 Zitolo, A. *et al.* Identification of catalytic sites for oxygen reduction in iron- and nitrogen-doped graphene materials. *Nat. Mater.* **14**, 937-942 (2015).

23 Malko, D., Lopes, T., Symianakis, E. & Kucernak, A. R. The intriguing poison tolerance of non-precious metal oxygen reduction reaction (ORR) catalysts. *J. Mater. Chem. A* **4**, 142-152 (2016).

24 Barrio, J. *et al.* FeNC Oxygen Reduction Electrocatalyst with High Utilization Penta-Coordinated Sites. *Adv. Mater.* **35**, e2211022 (2023).

25 Wan, X. *et al.* Fe−N−C electrocatalyst with dense active sites and efficient mass transport for high-performance proton exchange membrane fuel cells. *Nat. Catal.* **2**, 259-268 (2019).

26 Ehelebe, K. *et al.* Benchmarking Fuel Cell Electrocatalysts Using Gas Diffusion Electrodes: Inter-lab Comparison and Best Practices. *ACS Energy Lett.* **7**, 816-826 (2022).

27 Konrad, E. *et al.* Evaluating Electrocatalysts at Relevant Currents in a Half-Cell: The Impact of Pt Loading on Oxygen Reduction Reaction. *J. Electrochem. Soc.* **166**, F1259 (2019).

28 Hossen, M. M., Artyushkova, K., Atanassov, P. & Serov, A. Synthesis and characterization of high performing Fe-N-C catalyst for oxygen reduction reaction (ORR) in Alkaline Exchange Membrane Fuel Cells. *J. Power Sources* **375**, 214-221 (2018).

29 Poynton, S. D. *et al.* Preparation of radiation-grafted powders for use as anion exchange ionomers in alkaline polymer electrolyte fuel cells. *J. Mater. Chem. A* **2**, 5124-5130 (2014).

30 Firouzjaie, H. A. & Mustain, W. E. Catalytic Advantages, Challenges, and Priorities in Alkaline Membrane Fuel Cells. *ACS Catal.* **10**, 225-234 (2019).

31 Omasta, T. J. *et al.* Beyond catalysis and membranes: visualizing and solving the challenge of electrode water accumulation and flooding in AEMFCs. *Energy Environ. Sci.* **11**, 551-558 (2018).

32 Huang, G. *et al.* Composite Poly(norbornene) Anion Conducting Membranes for Achieving Durability, Water Management and High Power (3.4 W/cm2) in Hydrogen/Oxygen Alkaline Fuel Cells. *J. Electrochem. Soc.* **166**, F637-F644 (2019).

33 Nösberger, S. *et al.* The gas diffusion electrode setup as a testing platform for evaluating fuel cell catalysts: A comparative RDE‐GDE study. *Electrochem. Sci. Adv.* **3**, e2100190 (2022).

34 Ehelebe, K. *et al.* Fuel cell catalyst layer evaluation using a gas diffusion electrode half-cell: Oxygen reduction reaction on Fe-N-C in alkaline media. *Electrochem. Commun.* **116**, 106761 (2020).
35 Ku, Y.-P. *et al.* Impact of Carbon Corrosion and Denitrogenation on the Deactivation of Fe–N–C Catalysts in Alkaline Media. *ACS Catal.* **14**, 8576-8591 (2024).
36 Kellner, S. *et al.* The influence of commercial Ionomers and Membranes on a PGM-free catalyst in the Alkaline Oxygen Reduction. *ChemRxiv* (2024).
37 Mazzoli, L. *et al.* Inducing porosity in xylose-derived FeNC electrocatalysts for alkaline oxygen reduction. *Green Chem.* **26**, 3271-3280 (2024).
